# Ectopic sebaceous glands of the esophagus presenting as sessile polyps

**DOI:** 10.1055/a-2715-4980

**Published:** 2025-10-21

**Authors:** Kuan-Wei Liu, Sheng-Lei Yan

**Affiliations:** 1 Division of Gastroenterology, Department of Internal Medicine, Chang Bing Show-Chwan Memorial Hospital, Lugang Township, Changhua County, Taiwan

Ectopic sebaceous glands (ESGs) of the esophagus are very rare lesions, typically discovered incidentally during endoscopic examinations 1 2 . In 1978, Ramakrishnan and Brinker reported the first case of esophageal ESGs identified via endoscopy 3 . Esophageal ESGs were found in 0.05% of asymptomatic subjects in a study involving a population undergoing gastric cancer screening 2 . Most reported patients with esophageal ESGs were either asymptomatic or presented with symptoms of gastroesophageal reflux disease (GERD 1 2 4 ). Endoscopically, esophageal ESGs may appear as yellowish patches, plaques, or elevated lesions of varying sizes 1 2 4 . Although esophageal ESGs can be found throughout the esophagus, they were most commonly located in the middle and lower thirds 2 4 . We report here a new case of esophageal ESGs that presented as multiple sessile polyps, with the diagnosis confirmed by histopathological examination.

A 44-year-old man presented to our institution with worsening symptoms of acid regurgitation following the consumption of a fatty meal and alcohol. His medical history was notable for alcoholic fatty liver disease and GERD. Upper endoscopy revealed a sessile polyp in the middle esophagus ( Video 1 and Fig. 1 ), measuring approximately 0.4 cm in length. The lesion appeared semitransparent, with multiple small whitish pellets along its border. Additional smaller sessile polyps with similar endoscopic features were identified in the lower esophagus ( Fig. 2 ). Due to the uncertain nature of the lesions, biopsy specimens were obtained. Histopathological examination revealed polygonal cells with small central nuclei and abundant clear, granular cytoplasm containing foam-like fat droplets, located within relatively normal squamous epithelium and lamina propria ( Fig. 3 ). Immunohistochemical staining showed positivity for CK ( Fig. 4 ) and p40 ( Fig. 5 ), while immunostains for mucin and CD20 were negative. These findings were consistent with a diagnosis of ESGs. The patient remained under follow-up at our institution following the upper endoscopy examination.

Endoscopic video showing a sessile polyp in the middle esophagus. The polyp appeared semitransparent, with small whitish pellets along its border. Similar sessile polyps were identified in the lower esophagus.Video 1

**Fig. 1:**
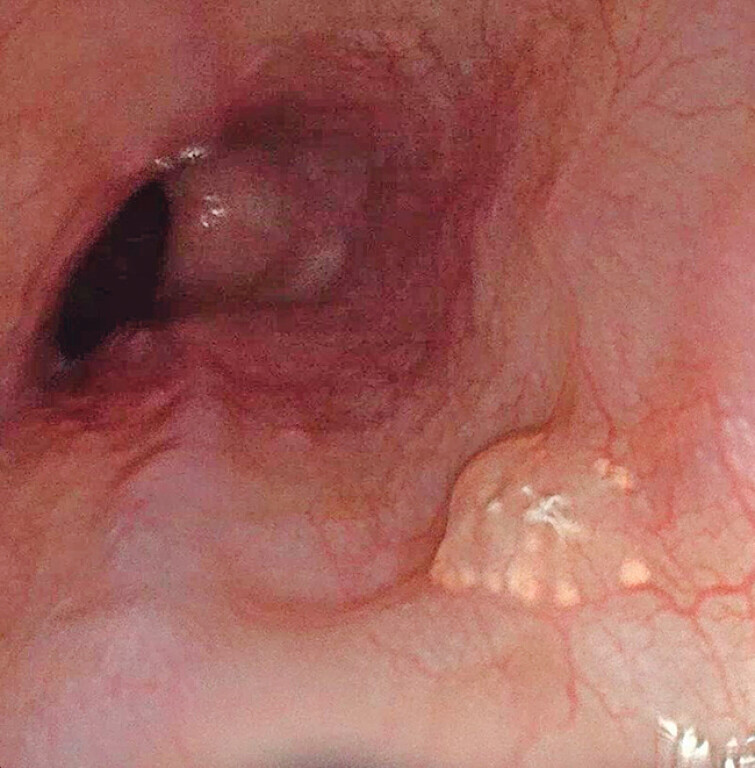
Endoscopic view showing a sessile polyp in the middle esophagus. The polyp appeared semitransparent, with multiple small whitish pellets along its border.

**Fig. 2:**
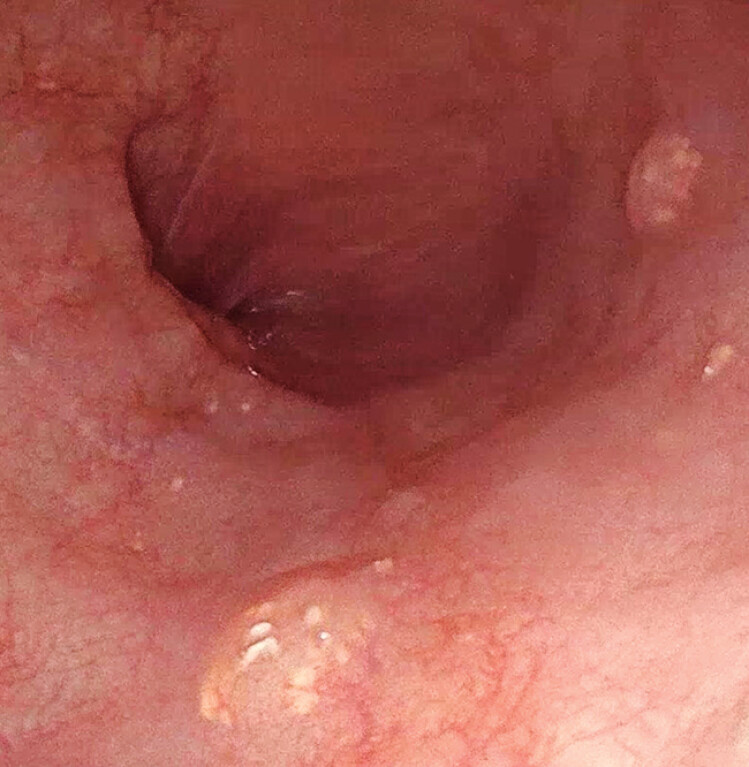
Smaller sessile polyps with similar endoscopic features were identified in the lower esophagus.

**Fig. 3:**
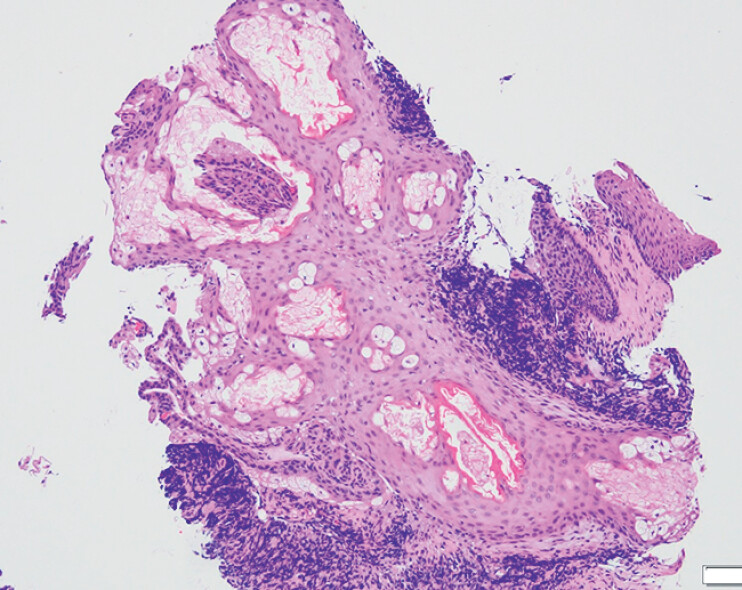
Photomicrograph showing polygonal cells with small central nuclei and abundant clear, granular cytoplasm containing foam-like fat droplets, located within relatively normal squamous epithelium and lamina propria (hematoxylin and eosin, magnification ×100).

**Fig. 4:**
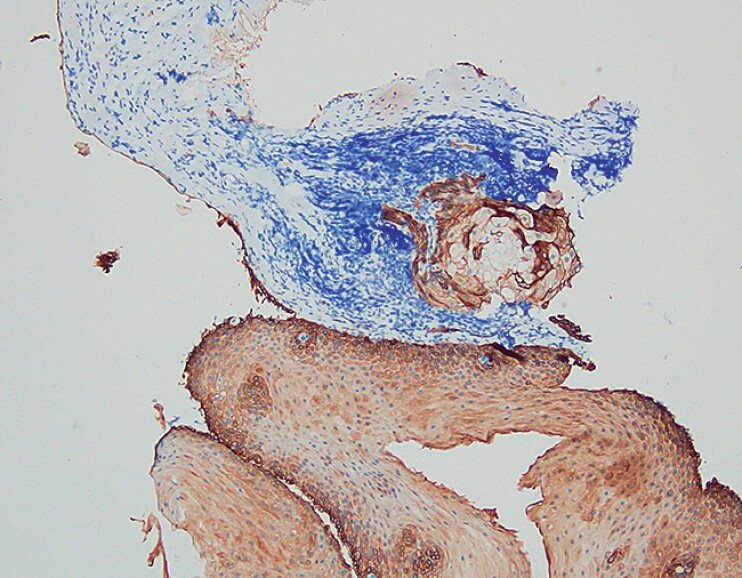
Immunohistochemical staining revealed positivity for CK (magnification ×100).

**Fig. 5:**
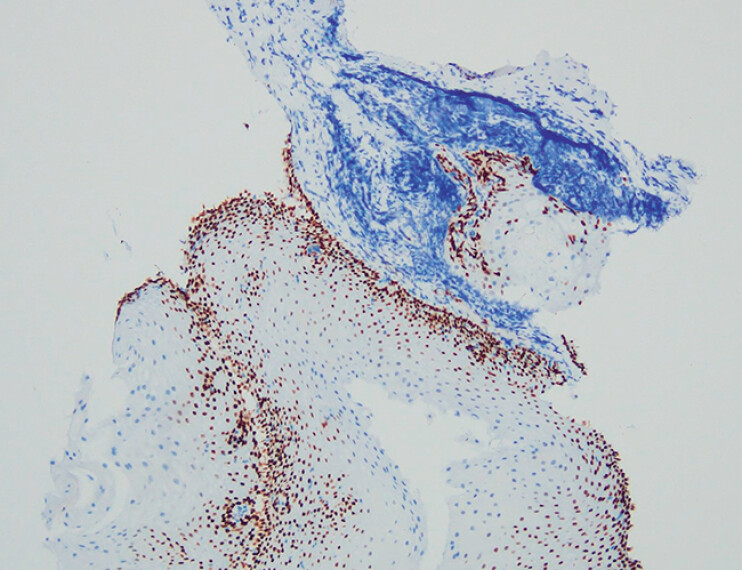
Immunohistochemical staining revealed positivity for p40 (magnification ×100).

Endoscopy_UCTN_Code_CCL_1AB_2AC_3AH

## Notes

Endoscopy E-Videos https://eref.thieme.de/e-videos
*E-Videos* is an open access online section of the journal *Endoscopy* , reporting on interesting cases and new techniques in gastroenterological endoscopy. All papers include a high-quality video and are published with a Creative Commons CC-BY license. Endoscopy E-Videos qualify for HINARI discounts and waivers and eligibility is automatically checked during the submission process. We grant 100% waivers to articles whose corresponding authors are based in Group A countries and 50% waivers to those who are based in Group B countries as classified by Research4Life (see: https://www.research4life.org/access/eligibility/ ). This section has its own submission website at https://mc.manuscriptcentral.com/e-videos .

## References

[1] Hashimoto H Horiuchi H Miura S Clinicopathologic Characteristics of Esophageal Ectopic Sebaceous Glands: Chronological Changes and Immunohistochemical Analysis Int J Surg Pathol 2021 29 378 384 10.1177/1066896920951844 32844680

[2] Park A Lee JH Park A Prevalence rate and clinical characteristics of esophageal ectopic sebaceous glands in asymptomatic health screen examinees Dis Esophagus 2017 30 1 5 10.1111/dote.12453 26822541

[3] Harada A Tatsumi Y Masumoto T Ectopic sebaceous glands Gastrointest Endosc 2004 60 97 15229434 10.1016/s0016-5107(04)01296-9

[4] Chen HF Lee HC Liao MK The clinical and endoscopic features of esophageal ectopic sebaceous glands Adv Dig Med 2020 7 179 187

